# Global pediatric research investigator: Heba M. S. El Zefzaf

**DOI:** 10.1038/s41390-023-02660-9

**Published:** 2023-05-22

**Authors:** Heba Mohamed-Sobhy El Zefzaf

**Affiliations:** Department of Pediatrics, Menoufia University, Shebin Elkom, Egypt


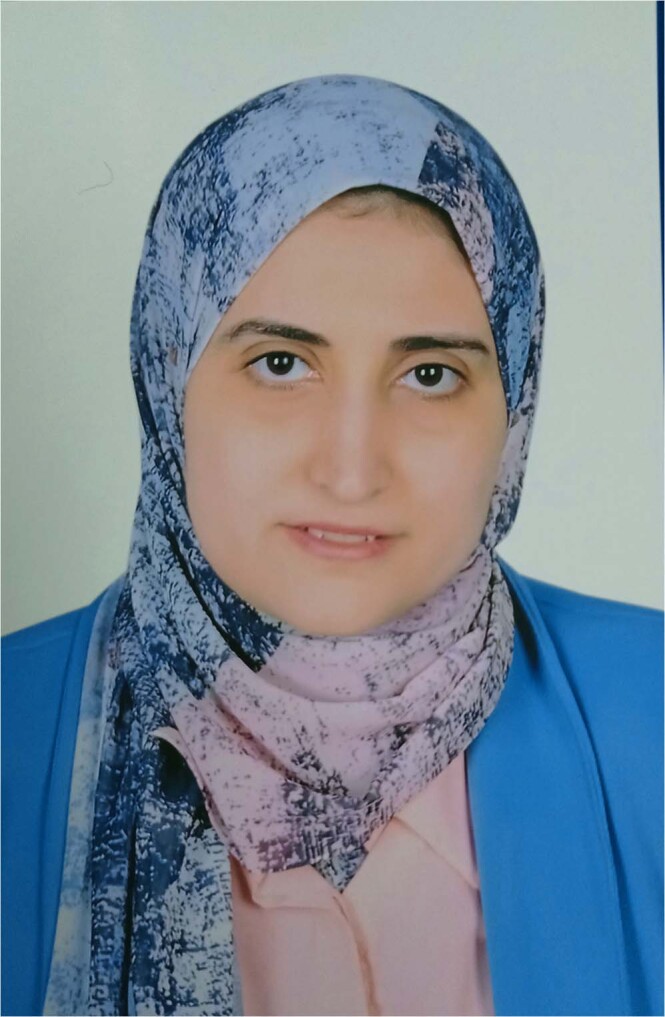


**GPRI: Dr / Heba Mohamed Sobhy El Zefzaf**

**Heba M.S. El Zefzaf**

I want to express my gratitude to the editor for giving me the opportunity to tell the readers of this illustrious magazine about the twelve years of my previous existence. I was born in the Egyptian city of El May in the Menoufia Governorate.

**My personal image**

I joined the Faculty of Medicine, Menoufia University, and specialized in pediatrics (Clinical nutrition, gastroenterology, and metabolism as a subspecialty). My first research was Evaluation of Cow’s Milk Related Symptom Score [CoMiSS] accuracy in cow’s milk allergy diagnosis and was published in pediatric research journal.

I was lucky and delighted to work with Professors Ali Mohamed. El-Shafie and Wael Abbas Bahbah, who encouraged and supported me to finish my research on the diagnostic efficacy of CoMiSS and recommended parameters to increase its efficacy in the diagnosis of cow’s milk allergy.

I appreciate Prof. Asmaa Abdel Sameea Mahmoud’s sound advice, which helped me to successfully complete my first research project. Also, I am grateful for the support I received from my husband Ahmed Shawky Abo Hola and my dear colleagues (Nahla Mahrous Al Sabbagh and Aya Abd El Razek Abu Hegazy).

I hope that after reading this overview, everyone will decide on a goal and work to achieve it because success in anything depends on having a cooperative and persistent attitude.

